# Screening of potential miRNA therapeutics for the prevention of multi-drug resistance in cancer cells

**DOI:** 10.1038/s41598-020-58919-2

**Published:** 2020-02-06

**Authors:** Zdravka Medarova, Pamela Pantazopoulos, Byunghee Yoo

**Affiliations:** MGH/MIT/HMS Athinoula A. Martinos Center for Biomedical Imaging, Massachusetts General Hospital and Harvard Medical School, Boston, MA 02129 USA

**Keywords:** Target identification, Cancer therapeutic resistance

## Abstract

Chemotherapy, a major cancer treatment approach, suffers seriously from multidrug resistance (MDR), generally caused by innate DNA repair proteins that reverse the DNA modification by anti-cancer therapeutics or trans-membrane efflux proteins that pump anti-cancer therapeutics out of the cytosol. This project focused on finding microRNAs that can regulate MDR proteins by managing corresponding mRNA levels through post-transcriptional regulation based on nucleotide sequence matching. Screening was done with bioinformatics databases for unpublished/unexplored microRNAs with high nucleotide sequence correspondence to two representative MDR proteins, MGMT (a DNA repair protein) and ABCB1 (an efflux protein), revealing microRNA-4539 and microRNA-4261 respectively. To investigate the enhancement of chemotherapeutics in cancer cells, high MGMT expressing glioblastoma (T98G) and a high ABCB1 expressing triple-negative breast cancer cell line (MDA-MB-231-luc) were treated with varying concentrations of chemotherapeutics and corresponding miRNAs. Newly identified MDR-related miRNAs (MDRmiRs) enhanced the response to anti-cancer therapeutics and resulted in effective cell death. In this study, we demonstrated that therapeutic miRNAs could be identified based on the nucleotide sequence matching of miRNAs to targeted mRNA and the same approach could be employed for the screening of therapeutic candidates to regulate specific target proteins in diverse diseases.

## Introduction

Cancer is the second leading cause of death worldwide, with approximately 42% of men and 38% of women being diagnosed with cancer in their lifetimes^1^. Patient treatment is mostly performed by surgical removal, chemotherapy, radiation therapy, or a combination of all three. Regardless of location in the body or stage of cancer progression, chemotherapy is considered a basic option for the treatment of cancer patients^2^. Chemotherapeutics modulate genomic or proteomic targets to eventually induce apoptosis in cancer cells. However, in many cases, cancer cells develop “multi-drug resistance” (MDR) against anti-cancer therapeutics during the course of chemotherapy^3^. Out of many MDR mechanisms, it is relatively well characterized that trans-membrane efflux proteins can pump the drug molecules out of the cancer cells to prevent the interaction with their targets, inducing multi-drug resistance, which is challenging to overcome in clinical anti-cancer treatment. Representatives of these efflux proteins include ATP-binding cassette subfamily B member 1 (ABCB1/MDR1), multidrug resistance-associated protein 1 (MRP1), and breast cancer resistance protein (BCRP/ABCG2)^4^. Specifically, ABCB1 is an ATP-dependent active transporter that regulates the efflux of chemotherapeutics from the cytosol with a broad spectrum of substrates, including hydrophobic drugs, peptides, lipids, steroids, protease inhibitors, and immuno-therapeutics^5^. In addition to the pumping-out of chemotherapeutics from the cytosol, MDR is induced by innate DNA repair systems^6^. In normal cells, the replication or transcription of DNA is inhibited by the addition of methyl groups (a type of alkyl group) to specific bases in nucleic acid side chains, while DNA repair systems work to remove the methyl groups for the revitalization of DNA^6^. Contrary to DNA intercalating chemotherapeutics with serious adverse effects, DNA alkylating agents lead to more effective cancer eradication with less side-effects^7^. However, this method suffers from the activation of DNA repair systems that repair the erroneous alkylation in DNA within a cancer cell. For example, Temozolomide, a DNA alkylating agent, is very effective for the treatment of primary glioma, but suffers from MDR due to the DNA repair function of O^6^-methylguanine methyltransferase (MGMT). MGMT removes methyl groups from DNA, allowing DNA to participate in transcription and cell proliferation once again^8^.

RNA interference is the phenomenon of mRNA degradation by the existence of complementary RNA sequences^9^. MicroRNAs (aka miRNAs) are small non-coding RNAs that can induce RNA silencing and post-transcriptional regulation of gene expression. MicroRNAs work together with the RNA-induced silencing complex (RISC) to deactivate mRNA. MicroRNAs can regulate specific mRNAs that are related to oncogenesis (oncomiRs), progression, metastasis (metastamiRs), as well as MDR (MDRmiRs), and they have pleiotropic effects on multiple pathways, physiological phenomena, and cell survival by targeting multiple mRNAs in a simultaneous manner^10^. By the RNA silencing mechanism, the RISC-microRNA association recognizes its target sequence within mRNA and deactivates mRNA by cleaving it at the translated region or the 3′-untranslated region (3′-UTR). On the other hand, siRNA sequences (synthetic) are designed to theoretically target the 3′-UTR consisting of complementary sequences with the exclusion of specific functional nucleic acid sequences^11^. Recently, web-based bioinformatics databases were constructed to provide the nucleotide sequences of different microRNAs, their tentative mRNA targets, sequence matching ratios, and related publications^12–15^. To demonstrate the hypothesis that microRNAs can be utilized for the prevention of MDR, microRNAs were screened through the bioinformatics databases and compared to those in former publications. Then, a list of MDRmiRs was identified as tentative therapeutics and finally, two microRNAs were selected with highest sequence matching to target mRNAs. In this study, we investigated the therapeutic effects of MDRmiRs for the enhancement of chemotherapeutics using a glioma cell line (T98G) with high MGMT expression and a triple-negative breast cancer cell line (MDA-MB-231-luc) with high ABCB1 expression.

## Methods

### Materials

The two microRNA mimics were provided by Integrated DNA Technology (IDT, Coralville, IA). The sequence of miR-4539 is 5′-S-S-GCUGAACUGGGCUGAGCUGGGC and the sequence of miR-4261 is 5′-S-S-AGGAAACAGGGACCCA. The 5′-end of the oligo was modified with disulfide capping to introduce a thiol group for conjugation with magnetic nanoparticles. The culture media, succinimidyl 3-(2-pyridyldithio)propionate (SPDP) and tris(2-carboxyethyl)phosphine (TCEP) were purchased from Thermo Fisher Scientific (Waltham, MA), while the fluorescent dye Cy5.5-NHS was purchased from GE Healthcare Life Sciences (Pittsburgh, PA). All antibodies for Western blotting were purchased from Abcam (Cambridge, MA). All other chemicals were purchased from Sigma-Aldrich (St. Louis, MO).

### Bioinformatics database search

Out of public, open source microRNA databases, Diana-TarBase, miRDB, miRbase, and microRNA.org were chosen for the quantitative screening of microRNAs that have complementary (matching) nucleotide sequences to the target mRNAs of chosen MDR proteins. Two major MDR proteins in cancer, O^6^-Methylguanine-DNA Methyltransferase (MGMT) and ATP Binding Cassette Subfamily B Member 1 (ABCB1), were chosen to be investigated for a complementary microRNA search^12–15^. The screening criteria of the miRNA sequences were chosen to be either matching nucleotide sequences in: (1) the 3′-UTR region in target mRNAs; or (2) the translated region sequence in target mRNAs. For this project, the thresholds for the lower boundary of effectiveness of the sequence matching were determined with a mirSVR score of −0.8 or lower, or a Target score of 80 or higher (with different score types being given in different databases). MiRNA candidates for each MDR mRNA were screened based on the screening thresholds. Each miRNA candidate was explored to confirm its relevance to cancer based on the prior literature. Finally, the miRNA hits that showed the highest score in the screened databases were selected for further investigation.

### Preparation of MN-miRNA

**Figure 1:**
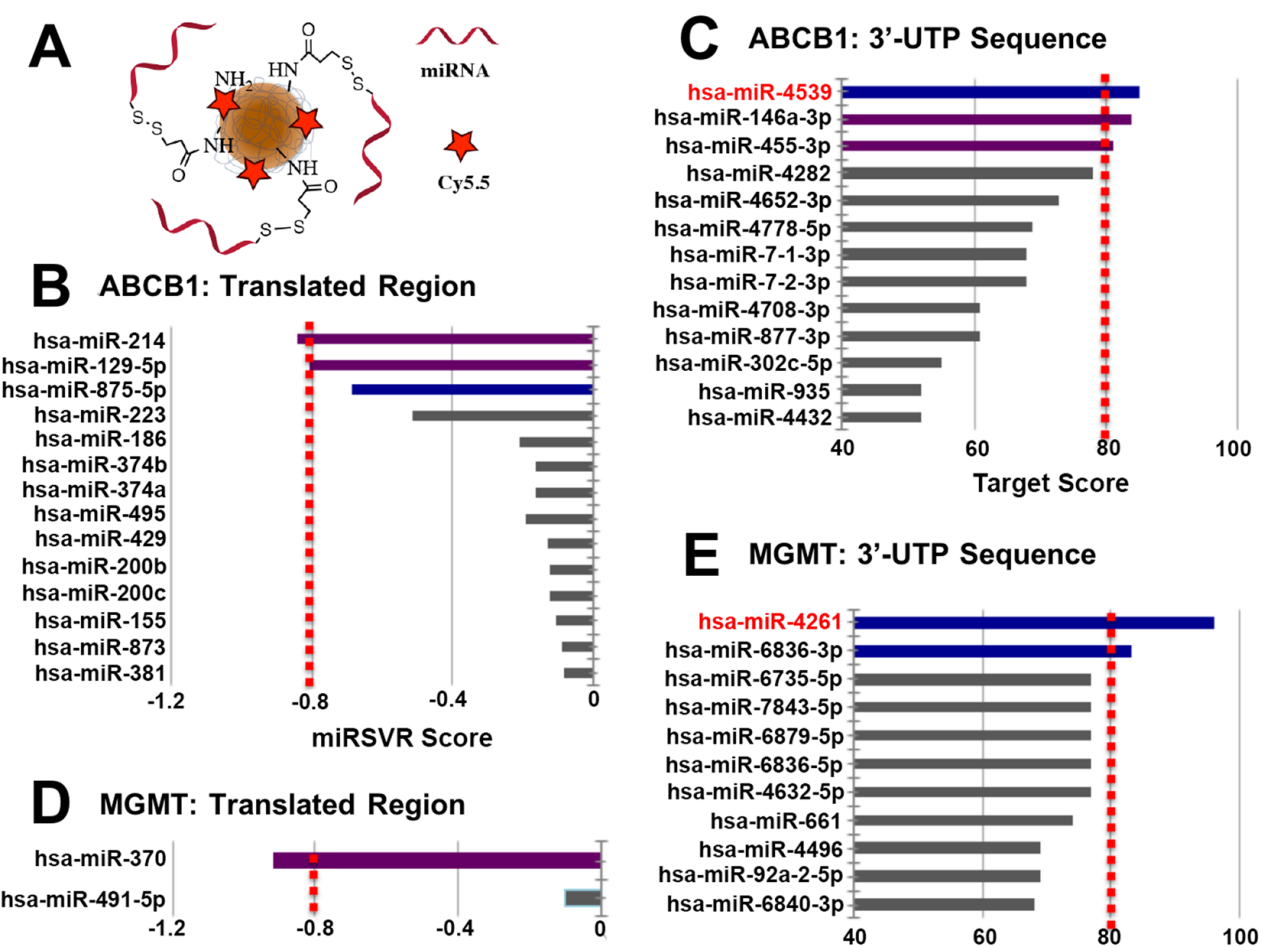
Screening of miRNA candidates for the inhibition of target MDR mRNAs. (**A**) Schematic of MN-miRNA. MN-miRNA consisted of dextran-coated iron oxide nanoparticles, conjugated to a near infrared (NIR) fluorescent dye (Cy5.5) and natural miRNA mimics, attached to the nanoparticles via a labile disulfide linker. (**B**) a list of miRNA that show complementary sequence matching to Translated region of ABCB1 mRNA, (**C**) summary of miRNAs that show complementarity to the 3′-untranslated region of ABCB1 mRNAs, (**D**) a list of miRNA that is complementary sequence matching to Translated region of MGMT mRNA, (**E**) summary of miRNAs that is complementary to the 3′-untranslated region of MGMT mRNA. MiRNA candidates for the regulation of MDR mRNAs were screened with a cut-off of −0.8 > miSVR (**B**,**D**) or Target Score > 80 (**C**,**E**). Blue/light blue: unreported, Purple: reported, Gray: lower than criteria.

Amine-derivatized iron oxide nanoparticles (MN-NH_2_) were prepared from dextran-coated iron oxide nanoparticles through modification with epichlorohydrin and ammonium hydroxide. The nanoparticles were labeled with Cy5.5, a near infrared (NIR) dye, by reacting 1 ml of MN-NH_2_ (120 μM, 14.4 mg Fe/ml) with 1 mg of Cy5.5-NHS ester (886 nmole, 7.5 eq.) in 100 μl DMSO. The reaction was carried out overnight at 4 °C, then the resulting Cy5.5-conjugated nanoparticles (MN-Cy5.5**)** were purified with a size exclusion column using nuclease free PBS buffer as an eluent. The MN-Cy5.5 was treated with excess amounts of SPDP (250 eq.) for 4 hrs at 4 °C to form MN(Cy5.5)-SPDP, which was again purified with a size exclusion column using nuclease free PBS buffer as an eluent. MC6 modified microRNAs were synthesized and provided by IDT (reference materials) and activated by TCEP to reduce disulfide bonds to thiol before conjugation. Activated miRNA-4539 and miRNA-4261 were combined with MN(Cy5.5)-SPDP to form MN-miR4539 and MN-miR4261, respectively (Fig. 1A). The molar ratio of miRNA to NP in conjugation was 10 to 1. The final products were stored in nuclease free PBS buffer at 4 °C until use.

### Characterization of MN-miRNA

The amount of Cy5.5 per nanoparticle was quantified by UV absorption at 675 nm (ε = 250,000). The number of SPDPs per nanoparticle was determined by quantifying pyridine-2-thion using UV spectrometer at 343 nm (ε = 8,080) after incubation of MN(Cy5.5)-SPDP with TCEP for 30 min. The iron content of the nanoparticles was determined by the Total Iron Reagent Set (Pointe Scientific, Canton, MI) using a UV spectrometer at 410 nm. Each experiment was performed three times. The amount of microRNA on MN-miR4539 and MN-miR4261 was determined by semi-quantitative agarose gel electrophoresis. Briefly, magnetic activated cell sorting columns (MACS, Miltenyi Biotech, Auburn, CA) were loaded with 5 μL of MN-miR4539 and MN-miR4261, respectively, and washed in a magnetic column with 100 μL of nuclease free water to remove unconjugated free microRNA. Pure MN-MDRmiR was collected by eluting the column with 20 μL of nuclease-free water. The collected MN-MDRmiR was treated with TCEP for 30 min to release miRNA from the MN. The TCEP-treated MN-MDRmiR, untreated MN-MDRmiR, and miRNA (50, 100, 150, 200 pmole) standard reference solutions were loaded onto a 2% agarose gel in Tris borate-EDTA buffer (Invitrogen, Carlsbad, CA) and electrophoresed at 120 V for 90 min. After electrophoresis, the microRNA residing in the gel was stained with 0.5 μg/ml of ethidium bromide for 30 min and visualized using a Molecular Imager FX scanner (Bio-Rad Laboratories, Hercules, CA). The intensities of miRNA bands in each image were quantified using ImageJ (ver.1.45i) software (NIH). The amount of miRNA released from the nanoparticles following TCEP treatment was determined relative to reference miRNA standard solutions. Relaxivities of nanoparticles were measured using a 20 MHz NMR spectrometer (miniSpec, Bruker, Billerica, MA).

The size of the magnetic nanoparticles (MN) was measured to be 19.87 ± 0.25 nm and increased up to 23.01 ± 0.82 nm after the addition of the fluorescent dye and miRNA. The number of amine groups per MN was found to be 56 (NH_2_/MN) by quantitative analysis of SPDP per MN. The ratio of fluorescent dye (Cy5.5) per MN was 3.3 (Cy5.5/MN). The average molar ratio of miRNA per MN was measured to be 5.7 ± 0.4 (miRNA/MN) by electrophoresis. Considering the reaction ratio of 10 miRNA per MN, the mean loading efficiency was calculated as 57%.

### Cell culture

The metastatic triple-negative human breast cancer cell line (MDA-MB-231-luc-D3H2LN (Perkin Elmer, Hopkinton, MA)), a cell line known to have the overexpression of ABCB1 proteins as a cause of MDR, and the glioblastoma multiforme cell line (T98G (ATCC)), a cell line known to have MGMT proteins as a cause of MDR, were cultured in Dulbecco’s modified Eagle’s medium, supplemented with 10% FBS (Thermo Scientific, Waltham, MA), 1% antibiotics (Invitrogen, Carlsbad, CA), and 2 mM L-glutamine, per the supplier’s instructions.

### Monitoring of *in vitro* uptake through fluorescence imaging

MN-miRNA uptake by the human cancer cell lines MDA-MB-231 and T98G was assessed through fluorescence imaging. T98G cells (ATCC) were seeded at a density of 1 × 10^5^ cells per well in a 12-well glass plate. MN-miR4261 was added into each well at various concentrations ranging from 0, 5 and 30 μM and incubated at 37 °C in a humidified 6% CO_2_ atmosphere for 48 hrs. The cells were fixed in 4% paraformaldehyde and mounted on slides with Vectashield Mounting Media with DAPI for nuclear staining. Microscopic images of Cy5.5 and DAPI were obtained using a Nikon Eclipse 50i microscope, and then raw images were imported by ImageJ and processed to generate an overlayed image, showing the subcellular location of the MN-MDRmiR. Following the same procedure, MDA-MB-231 was incubated with MN-miR4539 and the cellular uptake and localization of MN-MDRmiR in the cytosol was visualized following the same procedures described above.

### Quantification of MDR protein expression

The expression of the targeted MDR protein in each cell line was quantified by Western blotting after MN-MDRmiR treatment. Briefly, T98G cells were seeded in 12-well plates (1 × 10^5^ cells/well) and cultured for 24 hrs. The cells were incubated with 0, 5 and 30 μM of MN-miR4539 for 48 hrs without changing the media, then washed with HBSS. Next, the cells were harvested and treated with lysis buffer (a RIPA buffer with 100 mM EDTA, 100 mM PMSF and protease inhibitor cocktail). The amount of protein in the supernatant was quantified by a Pierce BCA assay (Thermo Scientific). Denatured protein extracts (40 μg) were loaded onto a polyacrylamide gel and proteins were separated through electrophoresis. After electrophoresis, the protein was transferred onto a polyvinylidene fluoride membrane, which was blocked in 5% non-fat milk for 1 hour. The membrane was incubated with rabbit monoclonal antibody to MGMT (dilution of 1:500) at 4 °C overnight and then labeled with secondary antibody (Goat anti-rabbit IgG (H + L), Dylight 488 pre-adsorbed, dilution of 1:400) for 1 hr at room temperature. The MGMT proteins were detected using the IVIS Spectrum imaging system (Perkin Elmer, Hopkinton, MA) and compared to reference β-actin that was detected using the same procedure. The expression of ABCB1 in MDA-MB-231 was quantified following the same procedure, but using MN-miR4539, the rabbit monoclonal antibody to ABCB1 (dilution of 1:500), and secondary antibody (Goat anti-rabbit IgG (H + L), Dylight 488 pre-adsorbed, dilution of 1:400).

### Cell viability

T98G cells (5 × 10^3^) were seeded on a 96 well plate (n = 3) and incubated for 24 hrs and treated with 1, 5 or 30 μM of MN-miR4261 and a varying concentration of Temozolomide (either 0, 0.2, 0.4, 0.6 or 0.8 μM). After 48 hrs, the cells were washed with HBSS, then 90 μL of the culture media and 10 μL of MTT solution (3-(4, 5-dimethylthiazol-2, 5-diphenyltetrazolium bromide, 5 mg/mL) were added to each well. These solutions stained the cells based on their metabolic activity. After 4 hrs of incubation, the cells were washed with DPBS twice, and suspended in DMSO. Each 96-well plate was measured at 570 nm with a reference of 630 nm to compare cell survival within each well.

### Statistical analysis

Data were expressed as mean ± s.d. or s.e.m., where indicated. Statistical comparisons were drawn using a two-tailed t-test (SigmaStat 3.0; Systat Software, Richmond, CA). A value of P < 0.05 was considered statistically significant.

## Results

### Screening of microRNA candidates for the regulation of ABCB1/MDR1 expression

From the database search, a number of microRNA sequences were found to show partial or significant sequence matching to ABCB1 mRNA. These miRNAs include hsa-miR-218, hsa-miR-186, hsa-miR-491-5p, hsa-miR-873, hsa-miR-520d-3p, hsa-miR-372, hsa-miR-373, hsa-miR-520c-3p, hsa-miR-520b, hsa-miR-106b, hsa-miR-519d, hsa-miR-499-5p, hsa-miR-411, hsa-miR-141, hsa-miR-200a, hsa-miR-651-5p, hsa-miR-5010-3p, hsa-miR-3925-5p, hsa-miR-6892-5p, hsa-miR-1200, hsa-miR-302e, hsa-miR-4721, hsa-miR-6763-5p, hsa-miR-7845-5p, hsa-miR-1296-3p, hsa-miR-520c-3p, hsa-miR-4760-5p, hsa-miR-135b-3p, hsa-miR-4539 and hsa-miR-548at-5p. After using the cutoff filtering criteria for sequence correspondence (mirSVR score < −0.8 or Target score > 80), six miRNAs (hsa-miR-214, hsa-miR-129-5p, hsa-miR-875-5p, hsa-miR-4539, hsa-miR-146a-3p and hsa-miR-455-3p) were identified as likely potential therapeutics for the regulation ABCB1 mRNA. From the literature search, a list of miRNAs was found to have already been investigated and reported, including miRNA-451, miRNA-129-5p, miRNA-7, miRNA-127, miRNA-196a, miRNA-508-5p, miRNA-145, miRNA-200c, miRNA-103, miRNA-107, miRNA-223, miRNA-381, miRNA-495, miRNA-455-3p, miRNA-214 and miRNA-146a^16–21^. Here, hsa-miR-214, hsa-miR-129-5p, hsa-miR-146a-3p, miRNA-455-3p and hsa-miR-875-5p were in both the list of six tentative therapeutics and the list of formerly investigated miRNAs. These miRNAs showed significant effects on the inhibition of multi-drug resistance or the suppression of cancer proliferation in diverse cancer types^21–26^. Considering the results in the former research, the six miRNA sequences found through the screening process used in this experiment were expected to induce significant suppression or prevention of ABCB1 protein expression and enhance the therapeutic efficacy of chemotherapeutics (Fig. 1B,C). Recently, miRNA-875-5p was explored as a key regulator of Epithelial-Mesenchymal Transition (EMT) in cancer metastasis^20,27,28^. However, it’s role in drug resistance has not been investigated yet. The high sequence matching of miRNA-875-5p to ABCB1 implied as high a possibility of the prevention of drug resistance as the former investigations about miRNA-495, -381, -146a, -146b, -214, -195 and -455^19,21,22,25,26^. Finally, only one miRNA sequence, hsa-miR-4539, was selected as a potential unexplored anti-cancer therapeutic for the regulation of ABCB1.

### Screening of miRNA candidates for the regulation of MGMT expression

**Figure 2:**
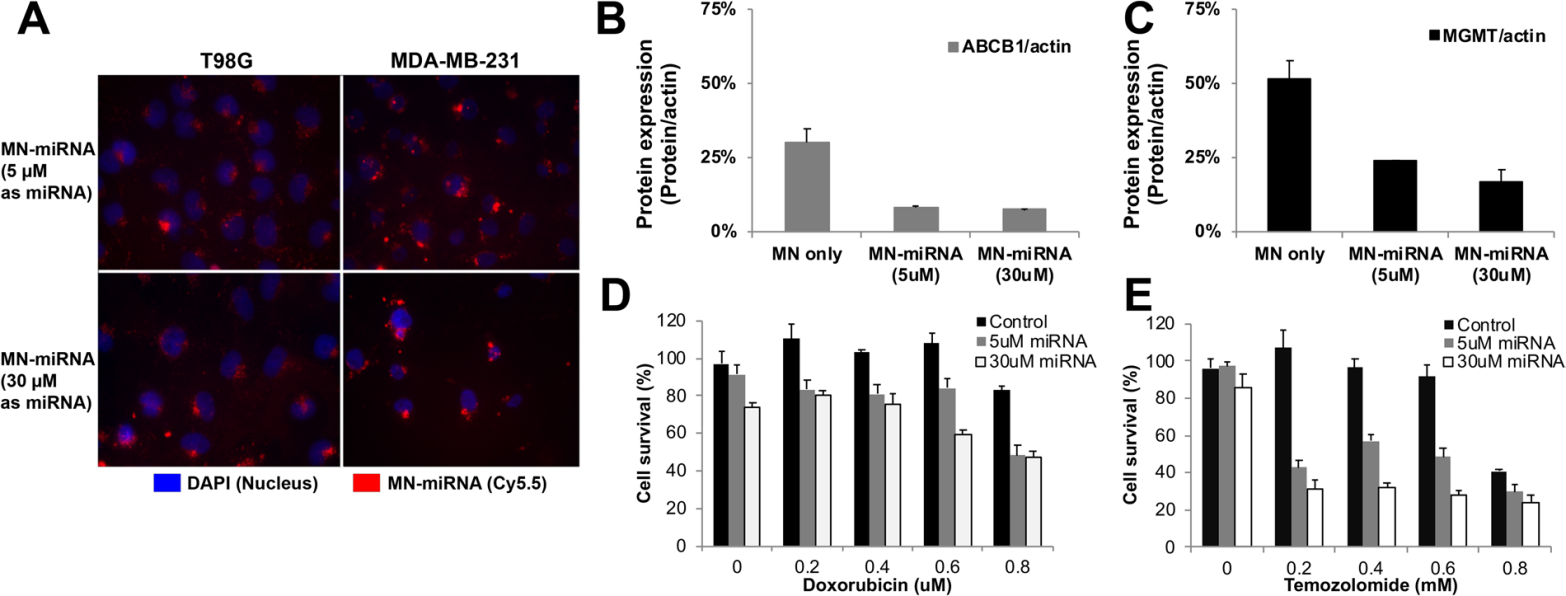
*In vitro* cell based experiments for the confirmation of therapeutic effects. (**A**) Fluorescence microscopy of cancer cells incubated with MN-miRNA. MN-miRNA was localized to perinuclear regions of the cell (Blue: DAPI, nucleus; Red: Cy5.5, MN-miRNA), (**B**) the quantification of ABCB1 expression level in MDA-MB-231 breast cancer cell with the treatment of MN-miR4539 by Western Blotting (the amount of ABCB1 was normalized by the amount of house keeping protein, actin), (**C**) the quantification of ABCB1 expression level in T98G glioblastoma multiforme with the treatment of MN-miR4261 by Western Blotting (the amount of MGMT was normalized by the amount of house keeping protein, actin), (**D**) quantification of cell viability following combination treatment with MN-miR4539 and doxorubicin (MDA-MB-231), (**E**) quantification of cell viability following combination treatment with MN-miR4261 and Temozolomide (T98G). Cell viability was normalized to an MN-only treatment. (*p < 0.05. Student’s t-test, n = 2).

From the database search, a number of miRNA sequences were found to show partial or significant sequence matching to MGMT mRNA. These miRNAs include hsa-miR-370, hsa-miR-491-5p, hsa-miR-4261, hsa-miR-6836-3p, hsa-miR-6735-5p, hsa-miR-7843-5p, hsa-miR-6879-5p, and hsa-miR-4436b-3p. By employing the same cutoff filtering criteria as ABCB1 (mirSVR score < −0.8 or Target score > 80), three miRNAs, hsa-miR-370, hsa-miR-4261 and hsa-miR-6836, were identified as potential therapeutics for the regulation of MGMT mRNA. From the literature search, a list of miRNAs was found to have already been investigated and reported, including hsa-miR-370, miRNA-181d, miRNA-767-3p, miRNA-648, miRNA-603, miRNA-221, and miRNA-222^29–37^. Here, hsa-miR-370 overlapped in both the list of the three tentative therapeutics and the list of formerly investigated miRNAs^31,38–40^. This miRNA showed significant suppression of cancer progression in diverse cancer types. Considering the results from miRNA-370, two miRNA sequences were expected to show significant therapeutic effects by the suppression or deactivation of MGMT and enhance the therapeutic efficacy of chemotherapeutics (Fig. 2C,D). Finally, two miRNA sequences, hsa-miR-4261 and hsa-miR-6836-3p, were selected as potential anti-cancer therapeutics for the regulation of the DNA repair protein, MGMT. In this study, hsa-miR-4261, which showed a higher Target score, was investigated further.

### Cellular uptake and subcellular localization of MN-miRNA

The cellular uptake and localization of Cy5.5-labeled MN-MDRmiR was monitored by fluorescence. As shown in Fig. 2A, MN-MDR-miR was localized in the perinuclear region consistent with intracellular trafficking via predominant lipid raft endocytosis, rather than clathrin-mediated endocytosis^41^. The cell density in the 5-μM miRNA treatment group was higher than that of the 30-μM miRNA treatment group in both cell lines, MDA-MB-231 and T98G.

### MN-microRNA induced down-regulation of target proteins

The change in the expression level of MDR proteins before and after MN-MDRmiR treatment was quantified by Western blotting. First, the triple negative breast cancer cell line, MDA-MB-231, was treated with MN only, then 5 μM of MN-miR4539, and lastly 30 μM of MN-miR4539. The expression of ABCB1 showed a significant dose-dependent decrease in response to treatment (Fig. 2B). ABCB1 expression was reduced by 67.59% after treatment with 5 μM of MN-miR4539 and 70.75% after treatment with 30 μM of MN-miR4539. The expression of ABCB1 protein was inhibited effectively even at a 5 μM concentration of MN-miR4539 (Fig. 2C). Similarly, the expression of MGMT in T98G cells was inhibited after treatment with MN-miR4261. MGMT protein expression was down regulated by 48.34% after treatment with 5 μM of MN-miR4261 and 72.41% with 30 μM of MN-miR4261. These results demonstrated that the MDRmiR treatment induced down-regulation of the target MDR proteins effectively.

### Combination treatment with MN-MDRmiR and anti-cancer therapeutics

To observe the potential of combined treatment with MN-MDRmiR and chemotherapy, we performed studies in which we co-administered MN-miR4539 and doxorubicin in the triple negative breast cancer cell line, MDA-MB-231 (Fig. 2D). Specifically, the concentration of doxorubicin was set as 0, 0.2, 0.4, and 0.8 μM, with the addition of varying concentrations of MN-miR4539 (0, 5 and 30 μM as miRNA). From the cell viability assay, no significant cytotoxicity was observed at concentrations of doxorubicin up to 0.8 μM. However, the cell viability was significantly affected by the co-administration of MN-miR4539. Up to 0.4 μM of doxorubicin, the cytotoxic effect was observed only with 30 μM of MN-miR4539 (p = 0.011) but not with 5 μM of MN-miR4539. In the case of 0.6 μM doxorubicin, cell viability was reduced to 84% with 5 μM MN-miR4539 (p = 0.00042) and 59% with 30 μM MN-miR4539 (p = 0.0015). When the concentration of doxorubicin was increased to 0.8 μM, the cell viability was reduced to 59% with 5 μM of MN-miR4539 (p = 0.0005) and 47% with 30 μM of MN-miR4539 (p = 0.0005) (Fig. 2D). Interestingly, with a low dose of doxorubicin, there was no significant difference in cell viability between 5uM and 30uM of MN-miR4539. This result indicated that the downregulation of MDR protein, ABCB1, was not sufficient to induce cytotoxic effects but enhanced the therapeutic efficacy of doxorubicin synergistically.

High MGMT expressing T98G glioblastoma cells were treated with varying concentrations of Temozolomide (0, 0.2, 0.4. 0.6 and 0.8 mM) (Fig. 2E). Up to 0.6 mM of Temozolomide, there was no significant change in the cell viability. However, the addition of MN-miR4261 reduced cell viability significantly, even at a Temozolomide concentration of 0.2 mM. When T98G cells were treated with 0.2 mM of Temozolomide and 5 μM of MN-miR4261, cell viability decreased to 42.8% of the Temozolomide only treatment. With the increase of concentrations, the cytotoxic effects of 30 μM MN-miR4261 showed a plateau at concentrations higher than 0.2 mM of Temozolomide. In contrast, co-administration of 5 μM MN-miR4261 induced additive effects in cell viability. Combined, these results indicated that the MN-miRNA mimics potentiated the effect of chemotherapy in both the glioblastoma and breast cancer cells through the downregulation of MDR proteins.

## Discussion

In this manuscript, we describe the identification of new miRNA candidates with relevance to multi-drug resistance in chemotherapy. Two representative MDR proteins were selected based on the key roles played by these effectors in the development of drug-resistance, representing an efflux pump or a DNA repair system. Presently, the screening of miRNAs has been mainly carried out through the analysis of variations in epigenetic profiles in cancer cells. In this approach, the function of irrespective miRNA could be overlooked and hidden behind the major effector miRNAs. We hypothesized that MDRmiR could be identified for the regulation of specific MDR proteins with the criteria of endogenous short non-coding RNAs, sequence matching with target mRNA, and a higher correspondence than mirSVR < −0/8 or Target score > 80, which are expected to be effective regulators for MDR mRNA. Endogenous miRNA could reduce the unexpected immune responses or off-targeting effects compared with exogenous RNA therapeutics, including siRNAs. We chose two representative MDR proteins, ABCB1 and MGMT, and screened tentative therapeutic MDRmiRs.

With an outlook towards therapeutic application of mimics corresponding to miRNAs, we utilized iron oxide nanoparticles (MN) as an image-guided delivery vehicle. MN is an ultrasmall iron oxide nanoparticle coated with amino-dextran with a size of 19.87 nm that is delivered with high efficiency to tumor cells *in vivo*, because of its long circulating time and enhanced accumulation through the EPR effect. It also permits image-guidance by MRI because it affects T2 relaxation times^42,43^.

In cancer cells, MN-MDRmiR efficiently engages the RNA-Induced Silencing Complex (RISC) and inhibits the expression of complementary mRNA targets^42^. A key advantage of this delivery approach derives from the fact that MN-MDRmiR incorporates a superparamagnetic nanoparticle core whose accumulation in tissues could be monitored by quantitative noninvasive MRI. This capability is highly significant when designing and optimizing therapeutic protocols in the process of drug development. In addition, MRI could be used during treatment to determine therapeutic concentration in tissue, which could be crucial for patients whose therapy is failing for unknown reasons.

Our results demonstrated efficient uptake of MN-MDRmiR by cancer cells and perinuclear localization that is consistent with the known localization of the RNA induced silencing complex (RISC) to perinuclear sites of RNA storage and processing. Also, the perinuclear localization of MN-MDRmiR implies delivery through predominant lipid raft endocytosis rather than clathrin-mediated endocytosis^41^.

The alignment of miRNA-4539 fell within the 3′-untranslated region of ABCB1 mRNA (Supplemental Fig. 1). There was inhibition of ABCB1 by MN-miR4539 but the effect was not dose-dependent, suggesting that the inhibition of ABCB1 could be compensated at lower doses of doxorubicin by other efflux pump proteins, such as ABCC1 (MRP1) and ABCG2 (BCRP). When MN-miR4539 was co-administered with doxorubicin, there was a dose-dependent decrease in viability independent of doxorubicin concentration. Doxorubicin alone showed no effect on cell viability at low concentrations (below 0.8 μM). At a doxorubicin concentration of 0.8 μM, cytotoxicity increased by 41.3% in the presence of 5 μM MN-miRNA4539 and 53.1% in the presence of 30 μM MN-miRNA4539. This result implies that in a future therapeutic scenario, co-treatment with miRNA4539 could permit the application of lower doses of doxorubicin to potentially minimize the associated adverse effects.

Our studies in a model of glioblastoma focused on MGMT. MGMT is a member of the innate DNA repair systems employed by the cell to correct erroneous DNA methylation. It is implicated in resistance to the anti-glioma chemotherapeutic, Temozolomide, which functions by methylating DNA base to inhibit its participation in cell division or transcription. MiRNA-4261 showed a highest sequence matching to MGMT mRNA and the expected alignment of miR-4261 fell within the 3′-UTR region of MGMT mRNA (Supplemental Fig. 2). In T98G cells, MN-miR4261 localized to perinuclear regions and inhibited the expression of MGMT in a dose dependent manner. The expression of MGMT was inhibited by 48.3% at a concentration of 5 μM MN-miRNA4261 and 72.4% at a concentration of 30 μM MN-miRNA4261. Cell viability showed a dose-dependent decrease with increasing concentrations of MN-miRNA4261 at all doses of Temozolomide. The combination treatment was highly effective, even at a 0.2 mM concentration of Temozolomide. Furthermore, above 0.4 mM Temozolomide, cell viability plateaued and was not further influenced by an increase in MN-miRNA4261 concentration. This suggests the existence of other compensatory mechanisms for resistance to Temozolomide in this cell line that limit the effect of MGMT inhibition. Although there are several DNA repair systems that have been identified, MGMT is known as the key player in removing methyl groups from DNA molecules. Therefore, it has been speculated that other DNA repair systems could make up the function of MGMT partially, but not completely substitute the role of MGMT^44^.

One potential limitation of the approach derives from the possibility that the mutation in the protein sequence of ABCB1 and/or MGMT in patient samples may lead to nonfunctional proteins and lack of target engagement and therapeutic efficacy. In that case, empirical knowledge about the specific sequence of the messenger RNAs in each individual patient will be necessary in order to finetune the rational design of the MDRmiRs. This approach is made more feasible by advancement in Next Generation Sequencing.

In summary, the present study identifies new miRNA candidates for the prevention of MDR caused by efflux protein and DNA-repair protein in breast cancer and glioblastoma. In addition, it describes the design of new nanoparticle-based miRNA mimics that are therapeutically relevant and could be applied to efficiently engage the endogenous RNA interference apparatus in tumor cells, following a simple intravenous injection^42,43,45^. If implemented in the clinic, this new approach can present a significant benefit to patient health by decreasing chemotherapeutic doses, and minimizing side effects, while increasing therapeutic efficacy.

## Supplementary information

Supplementary Information.
