# Supplementary material for: Screening of potential miRNA therapeutics for the prevention of multi-drug resistance in cancer cells
Source: Sci Rep. 2020 Feb 6;10:1970. doi: 10.1038/s41598-020-58919-2 (PMC7005303; doi:10.1038/s41598-020-58919-2)
Supplement: Supplementary file 1 — Supplementary Information. [file 41598_2020_58919_MOESM1_ESM.docx]

**Screening of potential miRNA therapeutics for the prevention of multi-drug resistance in cancer cells.**

Zdravka Medarova^*^, Pamela Pantazopoulos, Byunghee Yoo^*^

*MGH/MIT/HMS Athinoula A. Martinos Center for Biomedical Imaging, Massachusetts General Hospital and Harvard Medical School, Boston, MA 02129, USA.*

* Corresponding authors

Byunghee Yoo Ph.D., Instructor of Radiology

MGH/MIT/HMS Athinoula A. Martinos Center for Biomedical Imaging, Department of Radiology, Massachusetts General Hospital and Harvard Medical School, Boston, MA 02129, USA, Tel: 617-643-4889. Fax: 617-643-4865, E-mail: [byoo@mgh.harvard.edu](mailto:byoo@mgh.harvard.edu)

Zdravka Medarova, Ph.D., Associate Professor of Radiology

MGH/MIT/HMS Athinoula A. Martinos Center for Biomedical Imaging, Department of Radiology, Massachusetts General Hospital and Harvard Medical School, Boston, MA 02129, USA, Tel: 617-643-4889. Fax: 617-643-4865, E-mail: [zmedarova@partners.org](mailto:zmedarova@partners.org)

This work was supported in part by R01CA16346101A1 from the National Cancer Institute to ZM.

Conflict of Interest: Zdravka Medarova is Founder of TransCode Therapeutics, Inc.

Running title:

miRNA therapeutics for the prevention of drug resistance in chemotherapy

Key words:

microRNA, drug resistance, nanotechnology, RNA interference, DNA repair, ABC transporter


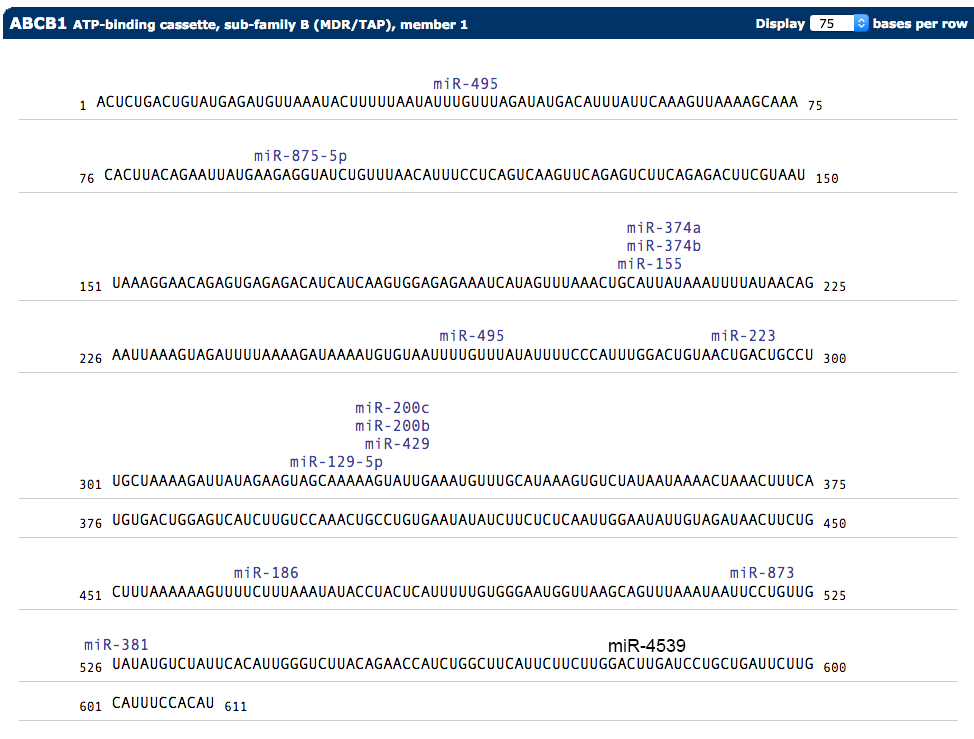


Supplemental Figure 1. Expected alignment of miRNA-4539 and ABCB1 mRNA. Additional miRNAs that showed alignment with the sequence of ABCB1 included miR-495, -875-5p, -374a, -374b, -155, -495, -223, -200c, -200b, -429, -129-5p, -186, -873, and -381. The figure was generated using the web-based miRNA database, miRNA.org.


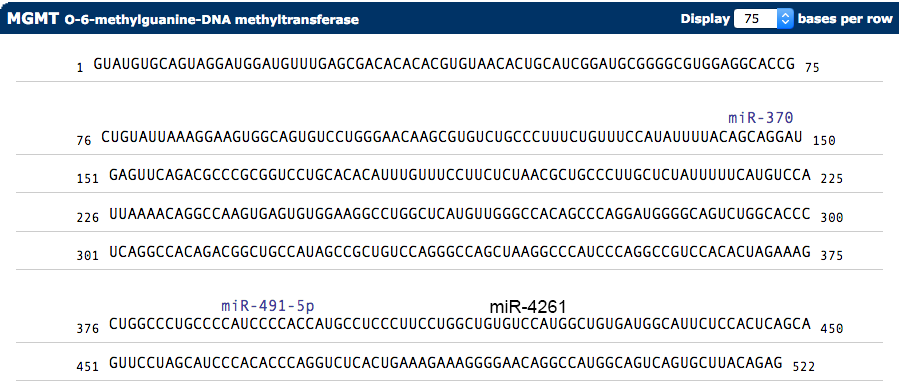


Supplemental Figure 2. Expected alignment of miRNA-4261 and MGMT mRNA. Only a few miRNAs showed alignment with the sequence of MGMT, including miRNA-370 and -491-5p. The figure was generated using the web-based miRNA database, miRNA.org.
